# Factors influencing accidental food allergic reactions in schools and preschools

**DOI:** 10.1007/s11845-023-03414-6

**Published:** 2023-06-07

**Authors:** Miranda Crealey, Aideen Byrne

**Affiliations:** 1 Department of Paediatric Allergy, Children’s Health Ireland at Crumlin, Dublin, Ireland; 2 Department of Paediatric Allergy, Children’s Health Ireland at Tallaght, Dublin, Ireland; 3 Department of Paediatrics and Child Health, Royal College of Surgeons in Ireland, Dublin, Ireland; 4 Department of Paediatrics, School of Medicine, Trinity College Dublin, Dublin, Ireland

**Keywords:** Allergic reaction, Anaphylaxis, Childcare, Food allergy, Preschool, School

## Abstract

**Background:**

Children spend a large proportion of their childhood in schools. In Ireland, there is no government policy on the management of food allergy (FA) in schools or preschool childcare settings (CCS). There is limited data worldwide on rate of accidental allergic reactions (AARs) within these settings.

**Aim:**

The aim of this paper is to report the management of FA and the incidence of AARs in Irish school or preschool CCS.

**Methods:**

A prospective observational study was established, enrolling children aged 2 to 16 years with confirmed FA. Participants were contacted at three monthly intervals for 1 year to report AARs to food. Data pertaining to schools and preschool CCS is reported here.

**Results:**

A total of 521 children (402 attending school and 119 attending preschool CCS) were enrolled. The annualised incidence of AARs in school was 4.5% (95% CI 2.6–7.0) and in preschool CCS 5% (95% CI 1.8–11.1); 6 of 7 of the nut reactions occurred in schools banning nuts. Half (3/6) of the preschool reactions were to cow’s milk; 174/521 (33%) children did not provide their individualised allergy action plan (AAP). Four out of 18 (22%) AARs in school were anaphylaxis and none were administered adrenaline by school staff.

**Conclusion:**

The incidence of AARs in this Irish cohort was found to be equivalent to the international experience. However, many of the recorded reactions identified in this study were likely avoidable. Preparation for AARs needs optimising. The ineffectiveness of “nut bans” remains unrecognised. Promoting milk and egg allergy resolution in infancy would likely reduce preschool- and school-based reaction numbers.

## Introduction

In Ireland, almost 5% of young children have a food allergy (FA) [1]. It can therefore be estimated that in every classroom or childcare room, there is at least one child with a FA. Children with FA are at risk of allergic reactions and anaphylaxis. There is no Irish data on the frequency of accidental allergic reactions (AARs) within educational facilities.

As well as planned daily episodes of food consumption in these facilities, there may be many unplanned episodes, e.g. birthday parties, bake sales, cultural holiday celebrations. This food is commonly brought in from outside, usually from the child’s home.

The cornerstones of FA management in schools and preschool childcare settings (CCS) include (i) methods to prevent relevant exposure to allergens and (ii) plans to recognise and treat allergic reactions and anaphylaxis [2]. Within the allergy clinic, parents and children are educated on and trained in the avoidance and management of allergic reactions. All families are given an (i) allergy action plan (AAP) (which details the recognition and management of a reaction) and (ii) letter (detailing the child’s allergy and the need for available treatment) to give to school and childcare staff. It is up to individual parents to both provide this information, to the school or childcare facility, and educate the staff on how to use this plan.

Currently, there is no national- or government-level food allergy policy (FAP) in Ireland within schools and preschool childcare settings. Each particular school or childcare facility has the ability to manage FA at their own discretion and may or may not have a FAP. This is in contrast with other countries where state- or countrywide legislation is in existence.

To be able to effectively safeguard children in school or childcare settings, we need to understand the current management of FA as well as the number of AARs occurring within these environments in Ireland. The main aims of this paper are to (i) report the rate of AARs in school and preschool CCS and their management and (ii) identify the routine practice with regards to FA management and prevention of AARs in school and preschool CSS facilities.

## Methods

A prospective observational study “Recording Accidental Allergic reactions in Children and Teenagers” (ReAACT) was established and participants were recruited from Children’s Health Ireland (CHI) at Crumlin or Tallaght. Data specifically relating to school and preschool CCS is reported in this paper. The term preschool CCS includes preschools, Montessori, play schools, nurseries, crèches, childminders, and other services looking after more than 3 preschool children [3].

To be enrolled, children had to satisfy the inclusion criteria: (i) age: ≥ 2 years but less than 17 years and (ii) have a confirmed diagnosis of immediate type IgE mediated FA to a common food allergen: cow’s milk, hen’s egg, peanut, tree nuts, fish, seeds.

Due to the high likelihood of frequent eczematous flares, contact reactions and reactions to new allergens among infants and toddlers < 2, it was decided that accidental reactions within this cohort would need to be studied separately. Participants were defined as having a diagnosis of IgE-mediated FA if either of the following 3 criteria were met at study entry: (i) a clear history of a recent reaction (previous 6 months) clinically consistent with immediate IgE-mediated allergy and a positive skin test > 3 mm or (ii) a history of a reaction in the past and a skin test in the past 6 months to that allergen, of > 7 mm or (iii) a positive food challenge performed at CHI Tallaght or CHI Crumlin in the past 6 months. This strict study inclusion criterion ensured that participants were food allergic at study entry.

Participants were recruited over 7 months (November 2018 to May 2019). Demographic, clinical and school/childcare attendance data was gathered. Once recruited into the study, participants were followed for 1 year for AARs. A single researcher contacted all participants at 3 monthly intervals by phone to collect information on AARs.

Reactions were graded as anaphylaxis or non-anaphylaxis using the National Institute of Allergy and Infectious Disease (NIAID) 2005 [4] clinical criteria. Ethics approval was received from the Research Ethics Committees (REC) in CHI at Crumlin (REC Reference: GEN/672/18) and CHI at Tallaght (SJH/TUH REC) [6]. Funding was provided from the National Children’s Hospital fund (grant award number 15119).

Descriptive statistics were compiled for all variables using SPSS version 27 (2020; SPSS Inc., Chicago, IL, USA). Demographic and clinical characteristics were compared using two-sample *t*-tests or Wilcoxon rank-sum tests for continuous variables and Fisher’s exact or chi-squared tests for categorical variables. The annual incidence rate of AARs was expressed as the number of events divided by the sum of the patients at risk. All tests were two-sided. A *P* value < 0.05 was considered statistically significant. Proportional differences between categorical variables were calculated by using relative risks (RR).

## Results

Within ReAACT, 521 participants attended school (*n* = 402: primary school *n* = 317, secondary school *n* = 85) and preschool CCS (*n* = 119: preschool *n* = 67, nursery *n* = 50) (Table 1).

**Table 1:** Children attending school and preschool childcare services

	**Overall** ***N* = 521** ***N* (%)**	**Preschool CSS** ***N* = 119** ***N* (%)**	**Primary** ***N* = 317** ***N* (%)**	**Secondary** ***N* = 85** ***N* (%)**
**Gender**				
Male	361 (69)	84 (71)	218 (69)	59 (69)
Female	160 (31)	35 (29)	99 (31)	26 (31)
**Mean age** (years) (SD)	7.6 (4)	3 (1)	8 (2)	14 (1)
**Number of food allergies**				
1 food allergy	174 (33)	33 (28)	107 (34)	34 (40)
> 2 food allergies	347 (67)	86 (72)	210 (66)	51 (60)
**Food allergens**				
Peanut	339 (64)	75 (63)	206 (65)	58 (68)
Treenut	258 (48)	41 (34)	164 (52)	43 (51)
Egg	187 (35)	50 (42)	109 (34)	28 (33)
Milk	79 (15)	19 (16)	51 (16)	9 (10)
Fish	50 (9.5)	11 (9)	30 (9)	9 (10)
Previous history of anaphylaxis*	130 (25)	20 (17)	79 (25)	31 (36)
**Attend afterschool facility**	42 (8)	N/A	42 (13)	N/A
**Type of preschool childcare service attended**				
Preschool/Montessori	67 (13)	67 (52)	N/A	N/A
Nursery	50 (10)	50 (39)	N/A	N/A
Childminder (4–5 children)	5 (1)	5 (4)	N/A	N/A
**Other childcare services used**				
Childminder (≤ 3 children)	18 (3)	7 (6)	11 (4)	N/A
Nanny	12 (2)	3 (3)	9 (3)	N/A
Relative	10 (2)	2 (2)	8 (3)	N/A

*CCS* childcare services, *SD* standard deviation

*A previous hx of anaphylaxis is defined as a history of anaphylaxis prior to study commencement

Table 1 illustrates the clinical and demographic details for the 521 participants.

### Availability of allergy management plans and policies

Overall, 323 (61%) had a FAP in place. Compared to preschool CSS and primary schools, secondary schools were less likely to have a FAP in place (RR 0.70, 95% CI 0.54–0.90, *P* = 0.0058). Two-thirds (66%) of parents had provided the facility with an AAP and this proportion was similar across all establishments (Table 2).

**Table 2:** Characteristics of food allergy management within each facility

	Overall *N* = 521	Preschool CSS *N* = 119 *N* (%)	Primary* N = 317 *N* (%)	Secondary** N = 85 *N* (%)
FA policy	312 (60)	61 (51)	210 (66)	37 (44)
Copy of AAP	347 (67)	79 (66)	210 (66)	57 (67)
AAIs brought to facility	510 (98)	116 (98)	313 (99)	82 (96)
AAI stored together	344 (65)	78 (66)	205 (66)	56 (65)
Carry own AAI	25 (5)	0	5 (2)	20 (24)
Nut-free facility	385 (74)	107 (90)	247 (78)	32 (37)
Bring food in from home	470 (90)	106 (90)	283 (89)	77 (90)
Food made on site	131 (25)	55 (46)	27 (9)	64 (77)
Consumed food made onsite	99 (19)	42 (35)	21 (7)	43 (51)
Consumed food brought in by others	346 (66)	45 (38)	230 (72)	71 (83)

*AAI* adrenaline autoinjectors, *AAP* allergy action plan, *CCS* childcare services, *FAP* food allergy policy

*Primary school; **Secondary school

### Adrenaline autoinjectors

Overall, 510 of participants (98%) had 2 AAIs available to them in each facility, but in 35% of cases, the two devices were stored in separate locations.

### Restrictions and food bans

Overall, 74% (*n* = 398) of the facilities banned nuts. A significantly larger number of preschool CSS (*n* = 105, 90%) banned nuts as compared to secondary schools (*n* = 32, 37%) (RR 2.34, 95% CI 1.76–3.10, *P* < 0.0001) and primary schools (*n* = 247, 78%) (RR 1.13, 95% 1.0371–1.23, *P* = 0.0056).

### Food available within the facility

The source of food consumed varied across facilities (Table 2). Very small numbers of primary schools distributed food on a regular basis (*n* = 27, 9%). In contrast, 64 (77%) of secondary schools had a canteen providing food.

### Food consumption within the facility

#### Regular

Facility-prepared food was regularly eaten by 42 (35%) of those in preschool CCS and 43 (51%) of those in secondary schools.

#### Occasional food treats

Parents were asked whether their child would consume food brought into the facility by another child/teacher. According to their parents, 71 (83%) of secondary school adolescents, 230 (72%) of primary school children and 45 (38%) of preschool CCS would consume food brought in by teachers and other students.

Table 2 illustrates how food allergy is managed within the preschool and school settings.

### Accidental allergic reactions in schools and childcare

#### Schools

Eighteen (12%) of the total reactions recorded in school-aged children in ReAACT occurred at school giving an annualised incidence of AARs in school as 4.5% (95% CI 2.6–7.0). Schools were the third most common site for AARs after home and food establishments in the ReAACT study. Primary school-aged children (5–12 yrs., *n* = 16) were twice as likely to react compared to those in secondary school (13–16yrs, *n* = 2) (RR 2.1, 95% CI 0.50–9.1, *P* = 0.3). Six of the 7 reactions known to be caused by a nut occurred in schools where nuts were banned. Four of 18 (22%) were cases of anaphylaxis. All 4 school-based anaphylaxis cases occurred in primary school-aged children, with 3 receiving adrenaline. No adrenaline was delivered by school staff. Two were delivered by parents on their arrival and 1 by emergency department staff (Table 3).

**Table 3:** Details of accidental allergic reactions occurring in schools and preschool childcare settings

Reaction description	School (*n* = 18) *N* (%)		Preschool CCS (*n* = 6) *N* (%)	RR	95% confidence interval	*P* value
	Primary *n* = 16 (89)	Secondary *N* = 2 (11)				
Causative allergen						
Cow’s milk	2 (11)		3 (50)	0.22	0.04–1.02	**0.054**
Egg	4 (22)|		1 (16)	1.5	0.20–10.86	0.688
Peanut	1 (6)		0	1.1	0.05–24.07	0.949
Treenut	2 (11)		0	1.84	0.10–33.81	0.680
Any nut	4 (22)|		0	3.31	0.20–54.00	0.399
Unidentified	5 (28)		2 (33)	0.83	0.21–3.22	0.792
Type of exposure						
Ingestion	10 (56)		5 (84)	0.66	0.38–1.15	0.146
Contact	7 (39)		1 (16)	2.33	0.35–15.30	0.377
Unknown	1 (6)		0	1.26	0.05–27.82	0.882
Who gave the food to the child						
Consumed themselves	11 (61)		0	8.47	0.57–125.53	0.120
Teacher	1 (6)		6 (100)	0.05	0.008–0.37	**0.002**
Parent	2 (11)		0	1.84	0.10–33.81	0.680
Friend	2 (11)		0	1.84	0.10–33.81	0.680
**Associated activity**						
Bake sale	1 (6)		0	1.10	0.05–24.07	0.949
Pancake Tuesday^a^	1 (6)		1 (16)	0.33	0.02–4.54	0.410
Birthday party	2 (11)		0	1.84	0.10–33.81	0.680
**Severity**						
Non-anaphylaxis	14 (78)		5 (84)	0.93	0.60–1.44	0.755
Anaphylaxis	4 (22)|		1 (16)	1.57	0.22–10.99	0.644
Cause of reaction*						
Human error/failure to follow basic procedure	9 (50)		5 (84)	0.60	0.33–1.07	0.080
Did not read ingredients	4 (22)|		1 (16)	1.09	0.14–8.03	0.931
Did read ingredients/possible cross contamination	4 (22)|		0	2.73	0.16–44.89	0.480
Unsure	1 (6)		0	1.10	0.05–24.07	0.949
T**reatment received**						
Antihistamine	17 (94)		6 (100)	0.94	0.84–1.05	0.317
Adrenaline autoinjector	3 (17)		0	2.57	0.15–43.86	0.512
Inhaled bronchodilator	2 (11)		1 (16)	0.63	0.06–5.80	0.684
None	0		0			
**Person administering adrenaline**						
Parent	2 (11)		0	1.84	0.10–33.81	0.680
Teacher	0		0			
Healthcare professional in hospital	1 (6)		0	1.10	0.05–24.07	0.949
**Hospital treatment**	4 (22)		0	3.31	0.20–54.00	0.399

Only 2/18 school AARs occurred in secondary school; due to this small number, all school reactions were analysed together. All 4 anaphylaxis cases occurred in primary schools

*P* value < 0.005 is significant. Significant value is in bold

*AAR* accidental allergic reaction, *RR* relative risk

*Cause of reaction: An attempt was made to identify a cause for the reactions by asking parents a standard set of questions

^a^Pancake Tuesday is an annual celebration during which pancakes are eaten

#### Preschool CCS

Six reactions occurred among children who attended preschool CSS (Table 3) giving an annualised rate of reaction in preschool CSS as 5% (95% CI 1.8–11.1); 3/6 (50%) were due to cow’s milk and 2 to unidentified allergens. There was one case of anaphylaxis.

Table 3 describes the details of the 18 reactions in schools and the 6 reactions in the preschool CCS. Only 2/18 school AARs occurred in secondary school; due to this small number, all school reactions were analysed together.

### Factors associated with reactions

Children with an AAP in their school or preschool CSS had a significantly lower risk of an AAR when compared to those without (RR 0.2, 95% CI 0.09–0.47, *P* = 0.0002) (Table 4). There were more AARs among children who consumed food made on site (RR 1.75, 95% CI 0.74–4.11, *P* = 0.195) and in those who consumed food brought in by others (RR 2.13, 95% CI 0.86–5.26, *P* = 0.100).

**Table 4:** Associated factors for reactions in schools and preschool childcare services

	**AAR** ***N* = 24**	**No AAR** ***N* = 497**	**RR**	**95% CI**	***P* value**
Nut-free facility	18	367	1.04	0.42–2.57	0.925
FAP	13	299	0.72	0.33–1.55	0.404
AAP	8	339	0.20	0.09–0.47	**0.0002**
Food made on site in facility	8	123	1.48	0.65–3.39	0.344
Consumed food made on site	7	92	1.75	0.74–4.11	0.195
Consumed only food brought from home	5	127	0.77	0.29–2.03	0.605
Consumed food brought in by others	18	228	2.13	0.86–5.26	0.100

*P* < 0.05 was deemed significant. Significant value is in bold. Chi-squared tests were used

*AAP* allergy action plan, *AAR* accidental allergic reactions, *CI* confidence interval, *FAP* food allergy policy, *RR* relative risk

Table 4 describes the factors associated with reactions in the educational and childcare facilities.

## Discussion

This study provides important data on FA management and reaction rates within the school and preschool setting in Ireland, in a cohort of infants and children for whom diagnosis is confirmed and recommendations provided. The annualised risk of reaction was 4.5% in schools and 5% in preschool CCS and the overall rate of anaphylaxis was 0.9%. Thus, it can be stated that there is the potential for 1 in 20 Irish food allergic children to have an AAR of any severity and 1 in 110 to have anaphylaxis in the facility they attend each year.

Data from other countries has demonstrated similar or higher risk profiles with 5–20% of all AARs occurring in schools [5–8]. However, Ireland lags behind in establishing a unifying mechanism for risk reduction. A collaborative approach between healthcare professionals and education governing bodies is required to introduce a standardised FAP in schools and preschool CCS. Australia, Canada France and the USA all have established system and policies for AAR reduction [9–12]. The UK has recently published a “Model policy for allergy management at school” document [13]. It includes an example of a working FA policy and supports existing UK government statutory guidance.

This study reveals that reaction to cow’s milk is a risk for young children attending preschool CCS in Ireland. Cow’s milk (CM) was the implicated allergen in 3 of the 6 (50%) preschool CCS reactions. Other studies also report high percentage of reactions to CM in preschool settings: 34% [14], 60% [15]. Indeed, persistent CM is the leading cause of fatal anaphylaxis in school-going children [16]. The early promotion of milk tolerance by the use of milk ladders is clearly important in reducing this future risk of reactions in milk-allergic infants [17].

Our study is consistent with international data that widespread nut bans in Irish schools did not result in fewer reactions [18, 19]. Six of the 7 nut AARs occurred in schools that banned nuts on the premises. This risk of a reaction was not decreased in schools that were designated “nut free” (RR 1.04, 95% CI 0.42–2.57, *P* = 0.925). It is the author’s opinion that nut bans discourage training in “not sharing food” which is critical to the avoidance of AARs. Three-quarters of AARs recorded in this study occurred due to children sharing food or ingesting food brought in for occasional events such as birthday parties. Furthermore, a break to a routine including a celebration or a new staff member creates increased risk. Sicherer also found a quarter of school/day-care reactions occurred when there was an interruption to routine [20].

The large number of reactions highlights that school and preschool CCS staff are not prepared for such events. Key components to preparation should include a FAP, AAP for all allergic children and easy access to AAIs. Less than two-thirds of schools had a FAP, as reported by parents.

Despite the study cohort all receiving a personalised AAP from the allergy clinic, 34% of parents did not provide the school/preschool CCS with this. Previous studies across the world report rates of 15–79% of children without an AAP in school [15, 21, 22]. Parents should be advised and reminded at clinic appointment to provide their school/preschool CCS with an AAP on a yearly basis. This provides an opportunity to educate the school staff on FA and identify and mitigate potential risks. As allergists, we need to support parents in communicating with the school and preschool CCS.

Five of the 24 school/preschool CCS reactions met the criteria for anaphylaxis, but no child received adrenaline by a staff member. Indeed, among the 17/18 who received antihistamine in school, in only 5 cases, did a school staff member administer it suggesting an overall poor response to allergic reactions. The rate of AAI administration in schools is variable worldwide with studies quoting rates of 33% [20] and 100% [15]. The data shown here indicates there is a need for education and training on FA management in school and preschool CCS. Stock adrenaline autoinjectors in schools have been shown to be cost-effective in other countries [23] and their introduction should also be considered in Ireland.

## Conclusion

Irish children are having AARs in preschool CCS and schools at a rate that mirrors closely the international experience. Schools commonly continue to apply ineffective prevention models such as “nut bans” at the risk of reduced focus on prevention of food sharing. Overall preparation for AARs in the form of individual AAPs for every food-allergic child appeared lacking. Timely recognition of the need for adrenaline needs to be taught to all who care for children with FA. The data collected here will inform any future development of national FA management policy.
